# Deep learning for time-series segmentation of mechanical ventilator waveforms

**DOI:** 10.1038/s41598-026-58565-0

**Published:** 2026-06-28

**Authors:** Preeti Gupta, Aditya Nemani, Virginia R. de Sa, Alex K. Pearce, Shamim Nemati, Atul Malhotra, Jason Y. Adams

**Affiliations:** 1 Scripps Research Translational Institute, San Diego, CA USA; 2 University of California San Diego, San Diego, CA USA; 3 University of California Davis, Davis, CA USA

**Keywords:** Computational biology and bioinformatics, Engineering, Health care, Mathematics and computing, Medical research

## Abstract

Accurate segmentation of ventilator waveforms is essential for detecting patient–ventilator asynchronies (PVAs), yet current heuristic methods can fail in noisy, real-world data. We developed and validated a deep learning model using a one-dimensional attention-gated U-Net architecture to identify inspiratory and expiratory onsets in mechanical ventilation waveforms. The model was trained and tested on 9719 breaths from 33 patients and outperformed published rule-based methods, achieving F1 scores of > 0.99 for both inspiratory and expiratory onset detection within a 0.1-s tolerance window. Performance remained robust in asynchronous breaths (F1 ≥ 0.98). When applied to quantify PVAs, the model reproduced reference standard asynchrony frequencies with no significant differences, whereas heuristic methods produced large deviations. Gradient-weighted class activation maps suggest that the model leveraged a diverse set of waveform features to inform segmentation. This computationally efficient model enables highly-accurate, clinically timely waveform analysis and provides a foundation for scalable, reproducible assessment of ventilator–patient interactions.

**Supplementary Information:**

The online version contains supplementary material available at https://doi.org/10.1038/s41598-026-58565-0.

## Introduction

Invasive mechanical ventilation (MV) is a common life-sustaining intervention for patients with acute respiratory failure, neurologic deterioration, or airway compromise. Each year, more than 2.1 million adults in the United States receive MV, and approximately 40% of intensive care unit (ICU) admissions require ventilatory support^1–3^. Despite its widespread use, MV use is associated with a high mortality rate of 30–43%, reflecting not only the severity of underlying illness but also complications such as ventilator-induced lung injury and ventilator associated complications^4,5^.

An important and increasingly recognized contributor to adverse outcomes is patient–ventilator asynchrony (PVA), which occurs when the ventilator’s breath delivery is misaligned with the patient’s intrinsic respiratory effort^6^. Asynchronies arise from mismatched ventilator settings, such as when inspiratory and expiratory cycles begin too early or too late relative to patient demand. This mismatch can generate excessive tidal volumes, a known risk factor for ventilator-induced lung injury, and elevate transpulmonary pressure, increasing global lung stress and strain^7,8^. Beyond global injury, asynchronies may also worsen regional lung stress and strain. Pre-existing lung injury causes pressure to distribute unevenly across lung regions, while pendelluft, the redistribution of air from non-dependent to dependent lung regions driven by spontaneous respiratory effort, further exacerbates local overdistention and collapse^9^. Risk factors for PVA include time of the day, ventilator mode and prescribed ventilator settings such as tidal volume and positive end expiratory pressures^10^. Additionally, clinical factors including a history of smoking and diagnoses such as pneumonia, sepsis or acute respiratory distress syndrome have been associated with increased PVA incidence^11^.

To identify PVAs, the inspiratory and expiratory phases of the breath cycle must be distinguished^12^. While experts can do this visually, large-scale systematic analysis requires automation to be feasible. At a minimum, ventilator waveforms must be segmented into individual breaths, since this pre-processing step forms the foundation of even the most advanced deep learning models developed to date^13–15^. Many ventilators and middleware platforms used for data capture do not provide this segmentation directly, so it must be inferred from pressure and flow measurements. Prior studies have attempted to define phase onsets using heuristic rules, but such approaches could be prone to failure in real-world data, which are frequently affected by noise, artifact, and the very asynchronies they aim to detect^16,17^. To our knowledge, there is limited prior work that has systematically compared the performance of heuristic methods to machine learning methods for breath segmentation of invasive mechanical ventilation waveforms in real-world datasets enriched for asynchronous breaths.

Deep learning approaches may address the challenge of segmenting real-world data. U-Net convolutional networks were originally developed for biomedical image segmentation to reduce the requirement for a large quantity of annotated training samples^18^. These approaches have since been adapted for one-dimensional (1-D) signals such as audio waves^19^ and MV waveforms^20^. As such, we utilized a U-Net architecture to develop a deep learning model to segment inspiratory and expiratory phases of ventilator waveforms. We hypothesized that this model would outperform rules-based segmentation methods. We further hypothesized that our model would maintain robust performance in the often noisy signals typical of asynchronous breaths. Finally, we assessed how different segmentation methods influence the quantification of asynchronies.

## Methods

This study was reported in accordance with the Checklist for Artificial Intelligence in Medical Imaging (CLAIM) guidelines (Supplementary Table 1)^21^. Although developed for imaging applications, CLAIM was selected because segmentation of ventilator waveform time series has important methodological parallels with imaging segmentation tasks. In the absence of a dedicated reporting standard for artificial intelligence applied to physiologic signals, CLAIM provided an appropriate framework to promote rigor, transparency, and reproducibility.

### Data

This retrospective study was a secondary analysis of previously collected ventilator waveform data at the University of California, Davis Health and was approved by its institutional review board, with informed consent obtained for all participants^22^. All methods were performed in accordance with the relevant guidelines and regulations. Following data collection, the dataset was enriched for segments with high rates of PVA by selecting regions that showed waveform irregularity at low magnification^22^. Supplementary Fig. 1 shows examples of normal, asynchronous and artifact-laden breaths in the dataset with corresponding inspiratory and expiratory onset labels. Data was collected from Puritan Bennet 840 ventilators, using a serial port connected by a serial-USB null modem cable to a Raspberry pi microcomputer. The ventilator generated pressure and flow measurements at a rate of 50 Hz. The dataset consisted of 9719 breaths from 33 unique patients, that were deidentified prior to receiving the dataset. Ventilator modes present in the dataset were identified using a previously developed machine learning classifier^23^. Labels for PVAs (double triggers, breath stack or asynchrony not otherwise specified) were previously annotated by two pulmonary and critical care physicians. All data from this dataset were included in the analysis, and no missing data was present. Data pre-processing entailed creating timestamps every 0.02s for each measurement.

The reference standard for the start of each inspiration was provided as a discrete label by the ventilator, selected with the assumption that ventilator-generated breath onsets incorporate intrinsic knowledge of ventilator breath delivery. Since expiratory onsets were not available from the ventilator, we implemented a previously published algorithm to generate candidate expiratory onset points, providing a standardized initial estimate across all breaths^22^. These candidate expiratory onsets were plotted where the flow waveform crossed zero following the largest positive continuous area under the curve. An expert pulmonary and critical care physician then manually reviewed every candidate and confirmed or relabeled each as appropriate, using a custom graphical user interface (GUI) developed in Python (version 3.13.5, Tkinter library)^22^. The physician disagreed with the algorithmic assignment in approximately 15% of instances, typically in breaths where the algorithm marked the onset of patient expiratory effort rather than the ventilator’s transition to expiration, reflecting our goal to label the precise time point when the ventilator cycled from inspiration to expiration rather than when patient expiratory effort began^20^. Difficult or ambiguous cases were reviewed with senior physicians with more than a decade of experience in ventilator waveform research, to ensure accuracy and consistency. Reference standards were developed for the entire dataset prior to cohort generation.

The dataset was split by patient file into training (80%), validation (10%) and test (10%) cohorts. No patient was present in more than one cohort. Based on sample size calculations, detecting an increase in accurately segmented breaths from 70 to 80% with 80% power would require a minimum of 291 breaths in the test set.

### Baseline models

The deep learning model was compared to two baseline models. The first, termed zero crossings, labelled inspiratory onsets when flow crossed from negative to positive values and expiratory onsets when flow crossed from positive to negative values^17^. The second model, termed the derivative backtracking method, was adapted from a previously published heuristic^24^. Inspiratory onsets were identified by first locating flow values greater than 12 L/min, then stepping backward to the point where the flow derivative began to change. Expiratory onsets were determined by finding flow values less than − 5 L/min that remained negative for at least 25 milliseconds after the inspiratory onset, and then stepping backward using the first derivative to identify where the flow crossed zero or plateaued.

### Deep learning model: data preparation and architecture

**Fig. 1:**
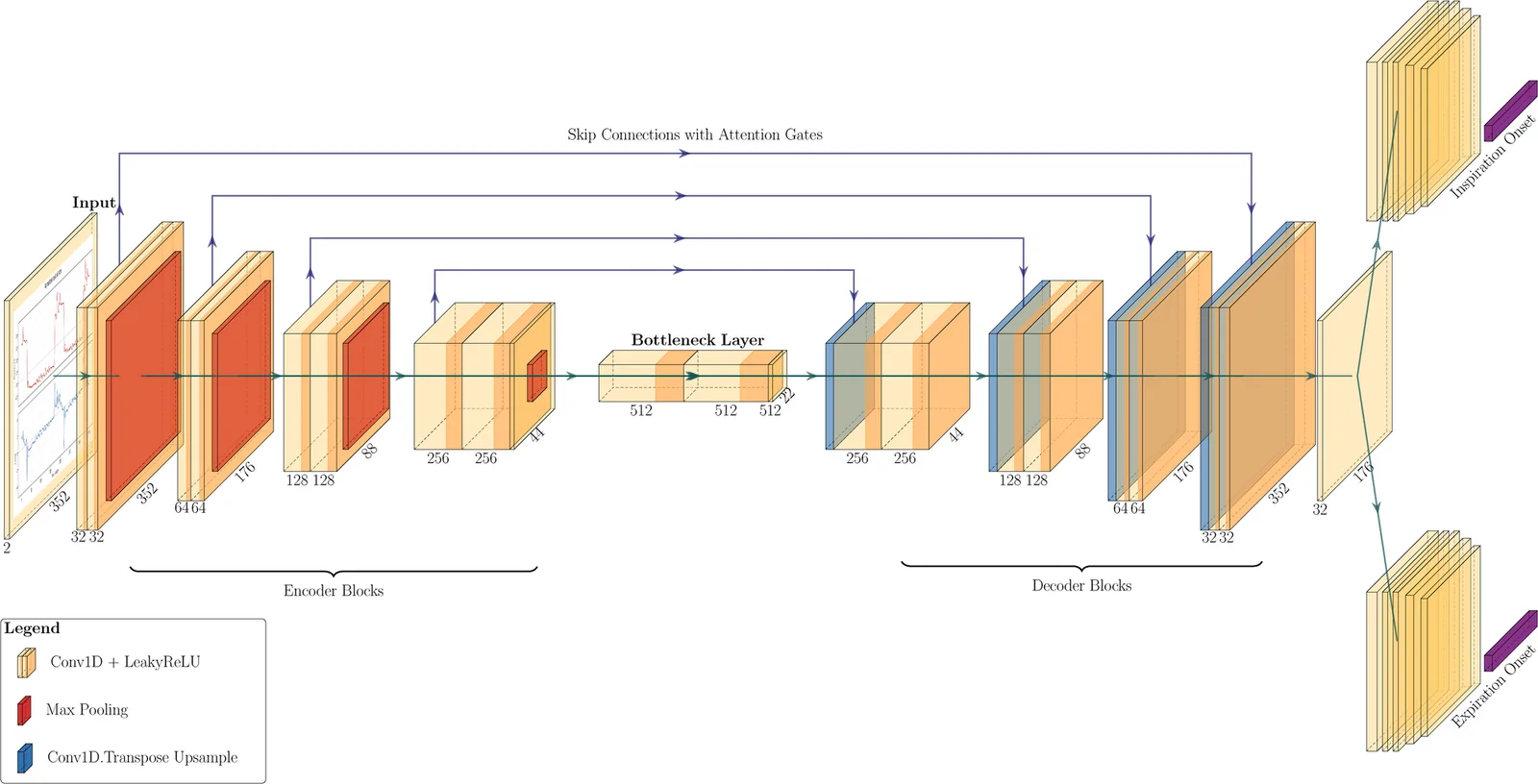
1D U-net with attention-gated skip connections for dual event detection. The diagram illustrates the four primary components of the model architecture: encoder blocks, bottleneck layer, decoder blocks, and two task-specific heads. The numbers below each block represent the size of the feature maps. The encoder comprises four convolutional blocks, with progressively doubling feature depth, each consisting of paired 1D convolution and LeakyReLU layers, and max pooling layers. The bottleneck uses dilated convolutions, LeakyReLU, and a dropout layer. The decoder mirrors the encoder using 1D transposed convolution upsampling blocks and concatenates the output with skip connections from the encoder, gated by attention mechanisms. This shared representation is center cropped and passed onto two task-specific heads: one for inspiratory onset and another for expiratory onset. Each 1D convolution layer of these heads is followed by layer normalization, dropout, and time distributed dense layers with GELU and a terminal sigmoid activation.

For the deep learning model, ventilator waveform data were segmented into fixed-length windows of 352 timesteps (7.04 s at 50 Hz), each containing two one-dimensional input channels: airway pressure and flow. For each input window, pressure and flow values were normalized so that each feature had a mean of zero and a standard deviation of one, ensuring that all signals were on the same scale before training. Each input window was paired with output labels spanning the central 176 samples (3.52 s, which corresponds to the average breath duration) to minimize edge effects. A two-channel one-dimensional convolutional neural network with a U-Net-style encoder–decoder architecture with attention-gated skip connections adapted from Oktay et al.^25^ was trained to jointly detect two types of cycling events, inspiratory and expiratory onsets, at each timestep in the output window (Fig. 1). The shared trunk then branches into dual heads, each specializing in its respective task. This design maintains efficiency through shared representations while enabling the model to capture idiosyncratic nuances in each head. This architecture also provides implicit regularization of the shared trunk, helping mitigate potential conflicts where certain weights and biases might benefit one task but hinder the other.

### Deep learning model training and post-processing

Training used an uncertainty-weighted composite loss combining focal and dice loss functions to address extreme class imbalance, event detection accuracy and dynamically balance the contributions of the two outputs^26^. This hybrid formulation was motivated by prior work demonstrating that Dice loss alone can be unstable under severe class imbalance and that combining Dice-based losses with cross-entropy-based terms may improve optimization stability in highly imbalanced segmentation tasks^27^. Specifically, the total loss was defined as:

$${\mathcal{L}}_{total}=\frac{1}{2{\sigma}_{1}^{2}}{\mathcal{L}}_{focal}+\frac{1}{2{\sigma}_{2}^{2}}{\mathcal{L}}_{dice}+\mathrm{log}{\sigma}_{1}+\mathrm{log}{\sigma}_{2}$$

where ${\sigma}_{1}$ and ${\sigma}_{2}$ are task-specific uncertainty parameters learned during training. The logarithmic terms act as regularizers that prevent ${\sigma}_{1}$ and ${\sigma}_{2}$ from diverging and help maintain numerical stability while balancing optimization across tasks. The focal loss was formulated as:

$${\mathcal{L}}_{focal}={-\alpha (1-{p}_{t})}^{\gamma} \; \mathrm{log}\left({p}_{t}\right)$$

where ${p}_{t}$ is the predicted probability of the true class, *α* is a balancing factor and *γ* controls the focusing strength, to diminish the influence of easily classifiable examples on the overall loss. The Dice loss was defined as:

$${\mathcal{L}}_{{dice}} = 1 - \frac{{2\sum\nolimits_{i} {p_{i} g_{i} } + \epsilon }}{{\sum\nolimits_{i} {p_{i} } + \sum\nolimits_{i} {g_{i} } + \epsilon }}$$

Here, ${p}_{i}$ denotes the predicted probability for element *i*, ${g}_{i}$ represents the corresponding ground-truth label, and *ε* is a small constant added for numerical stability. This function ensures better temporal alignment between predicted events and ground truth.

The model was initialized using He initialization and trained with the AdamW optimizer with an initial learning rate of 5e−4, mini-batches of 32 windows, dropout layers and early stopping based on validation loss (patience of 10 epochs, minimum delta of 1 × 10⁻⁴) to prevent overfitting^28^. The final model was selected as the epoch with the lowest validation loss under these criteria. Implementation was performed in TensorFlow/Keras and trained with GPU acceleration. During training, output windows were generated with a step size of 176 samples, resulting in non-overlapping output label spans. After inference, predictions were refined with debouncing and segment-level constraints, allowing at most one expiratory onset (with the maximum probability) per predicted breath interval, to improve clinical plausibility and reduce spurious detections. End-to-end computation time was measured per input window, and the mean (± standard deviation) total computation time was 348 (± 3.57) milliseconds per window.

### Evaluation

Due to class imbalance, with 0.7% of data points representing onset events, the F1 score, the harmonic mean of precision (positive predictive value) and recall (sensitivity), was chosen to be the primary evaluation metric. Unlike accuracy-based metrics, which are dominated by the large proportion of non-onset time points, the F1 score directly reflects the model’s ability to balance false positive and false negative onset detections, both of which can substantially bias downstream estimates of breath timing and patient–ventilator asynchrony. The model’s performance was evaluated against the reference standard in the test set both at the exact time point and within a 0.1 s tolerance window. This window was chosen based on prior literature showing that patients exhibit no conscious or unconscious respiratory responses to occlusion within this period, suggesting this is a clinically insignificant time period that would avoid interference with PVAs while still allowing a small tolerance in breath segmentation^29^. This window duration was further informed by an analysis of our dataset in which inspiratory onsets detected more than 100 milliseconds after the true onset produced over 10% error in estimated tidal volume, further supporting the use of this threshold as both physiologically and analytically appropriate (Supplementary Fig. 2). Additionally, to assess whether performance was driven by any individual patient, F1 scores within the 0.1 s tolerance window were calculated separately for each patient in the test set, and the mean and standard deviation were reported. To characterize model performance further, sensitivity analyses of the test set were performed by breath type (normal vs. asynchronous), based on the annotated dataset labels. Model interpretability was assessed by applying gradient weighted class activation maps (Grad-CAM)^30,31^ on manually selected input windows.

The secondary objective was to compare PVA quantification across the highest performing segmentation methods. Asynchrony frequency, defined as the ratio of asynchronous breaths to total breaths, was calculated using the segmentation reference standard, according to two published definitions for double triggering and one for stacked breaths^12,22^. The first definition (from Thille et al.) classifies a double-triggered breath as having an expiratory time less than 50% of the mean inspiratory time^12^. The other (from Adams et al.) defines a double-triggered breath as one with an expiratory time ≤ 0.3 s combined with either an expiratory-to-inspiratory tidal volume ratio (TVe/TVi) < 0.25, or a TVe/TVi < 0.50 with an expiratory tidal volume < 100 mL^22^. In contrast, for stacked breaths, the definition (from Adams et al.) requires an expiratory time > 0.3 s with TVe/TVi < 0.9^22^. Segmentation outputs from both the derivative backtracking method and the deep learning model were applied to the test set, and asynchrony frequencies were calculated accordingly. Statistical comparisons were performed using two-sided t-tests for continuous variables and chi-squared tests for categorical variables.

## Results

The deidentified dataset consisted of 9719 breaths from 33 patients who required various modes of MV for a range of acute indications. Approximately half of the breaths occurred in pressure control mode, with about 20% each in volume control and pressure support modes, and the remainder in proportional assist ventilation (Supplementary Table 2). The train and validation dataset contained 8520 breaths from 29 patients and the test dataset contained 1199 breaths from four patients, with no patient overlap between datasets (Table 1). The prevalence of PVA, as defined by expert annotators, was 28% in the training/validation dataset and 32% in the test dataset. Compared with the training/validation cohort, the test set was characterized by higher airway pressures and flows and shorter expiratory durations, which was an artifact of patient-level splitting.

**Table 1:** Cohort characteristics.

	Train and validation (*n* = 8520 breaths)	Test (*n* = 1199 breaths)
Mean pressure (cm H2O), median (IQR)	13.0 (10.3–16.7)	17.2 (15.2–21.9)
Peak pressure (cm H20), median (IQR)	25.2 (20.8–31.9)	31.4 (29.4–35.0)
Peak flow (L/min), median (IQR)	59.7 (50.4–70.5)	71.2 (60.6–84.1)
Tidal volume (mL), median (IQR)		
Inhaled	389 (280–494)	397 (328–452)
Exhaled	404 (278–512)	377 (304–456)
Inspiratory duration, s (IQR)	0.9 (0.8–0.9)	0.9 (0.8–0.9)
Expiratory duration, s (IQR)	1.8 (1.2–2.8)	1.6 (1.1–1.8)
Breaths/patient, n	294	300
Type of breath, n (%)		
Normal	5675 (67%)	815 (68%)
Asynchrony	2345 (28%)	380 (32%)
Artifact	500 (6%)	4 (0.3%)

### Model performance

The final deep learning model contained 8.9 million parameters (3.0 million trainable; ≈11 MB). At the native sampling resolution of 50 Hz, the deep learning model substantially outperformed both baseline approaches for detecting cycling events at the same sampled timepoint of the reference standard. For inspiratory onsets, the model achieved an F1 score of 0.981, compared with 0.561 for the derivative backtracking method and 0.061 for zero-crossing. For expiratory onsets, the deep learning model reached an F1 score of 0.937, whereas baseline methods performed with an F1 score of 0.009 and 0.011, respectively (Table 2). The heuristic methods generated substantially more candidate onsets than the reference standard, consistent with a high rate of false positive detections and low precision.

Within a tolerance window of ±0.1 s of the reference standard, the baseline models improved, but the deep learning model still exceeded their performance, achieving a recall of 0.996 and precision of 0.997 for inspiratory onsets. Recall was 0.995 and precision 0.984 for expiratory onsets (Table 2). In contrast, within this 0.1 s margin of error for inspiratory onset, the zero-crossing method and the derivative backtracking method achieved low precision of 0.546 and 0.698, respectively. Similarly, for expiratory onsets within the 0.1 s margin of error, the zero-crossing model had a precision of 0.474 and the derivative backtracking method had a precision of 0.708. The heuristic models had lower recall for expiratory onsets than inspiratory onsets. Per-patient analysis in the test cohort showed consistent performance across patients (Supplementary Table 3). Additional validation-set experiments comparing alternative loss functions and attention-gated versus non-gated U-Net architectures, performed during model development, are provided in Supplementary Table 4.

**Table 2:** Onset detection at native sampling resolution (50 Hz) and within a ± 0.1s Tolerance window.

	Model (number of onsets)	F1 score	Precision	Recall	F1 score (within 0.1 s)	Precision (within 0.1 s)	Recall (within 0.1 s)
Inspiratory onset (True *n* = 1199)	Deep Learning (*n* = 1198)	0.981	0.982	0.981	0.996	0.997	0.996
	Zero Crossing (*n* = 1940)	0.061	0.049	0.079	0.675	0.546	0.883
	Derivative Backtracking (*n* = 1561)	0.561	0.496	0.646	0.789	0.698	0.908
Expiratory onset (True *n* = 1198)	Deep learning (*n* = 1211)	0.937	0.932	0.942	0.990	0.984	0.995
	Zero crossing (*n* = 1937)	0.009	0.007	0.011	0.586	0.474	0.766
	Derivative backtracking (*n* = 1204)	0.009	0.009	0.009	0.710	0.708	0.712

s = seconds.

### Performance in normal and asynchronous breaths

When stratified by type of breath, the deep learning model also outperformed the best baseline heuristic method (Table 3). For normal breaths, the model achieved an F1 score of 0.994 within 0.1 s of inspiratory onsets and 0.995 within 0.1 s of expiratory onsets, compared to 0.845 and 0.685 for the derivative backtracking method, respectively. In breaths annotated as PVAs, deep learning model performance remained high, with F1 scores of 0.992 for inspiratory onsets and 0.979 for expiratory onsets. In contrast, the derivative backtracking method demonstrated a decline in performance for inspiratory onset detection (F1 score 0.629), while showing a slight improvement for expiratory onsets (F1 score 0.757).

**Table 3:** Subgroups by type of breath (normal vs. PVA).

	F1 (within 0.1 s)	Precision (within 0.1 s)	Recall (within 0.1 s)
Normal breaths (*n* = 815)			
Inspiratory onset			
Deep learning	0.994	0.995	0.993
Derivative backtracking	0.845	0.813	0.880
Expiratory onset			
Deep learning	0.995	0.994	0.996
Derivative backtracking	0.685	0.699	0.672
PVA breaths (*n* = 380)			
Inspiratory onset			
Deep learning	0.992	0.990	0.995
Derivative backtracking	0.629	0.492	0.871
Expiratory onset			
Deep learning	0.979	0.967	0.992
Derivative backtracking	0.757	0.724	0.794

*s* seconds, *PVA* patient-ventilator asynchrony.

### Model interpretability

**Fig. 2:**
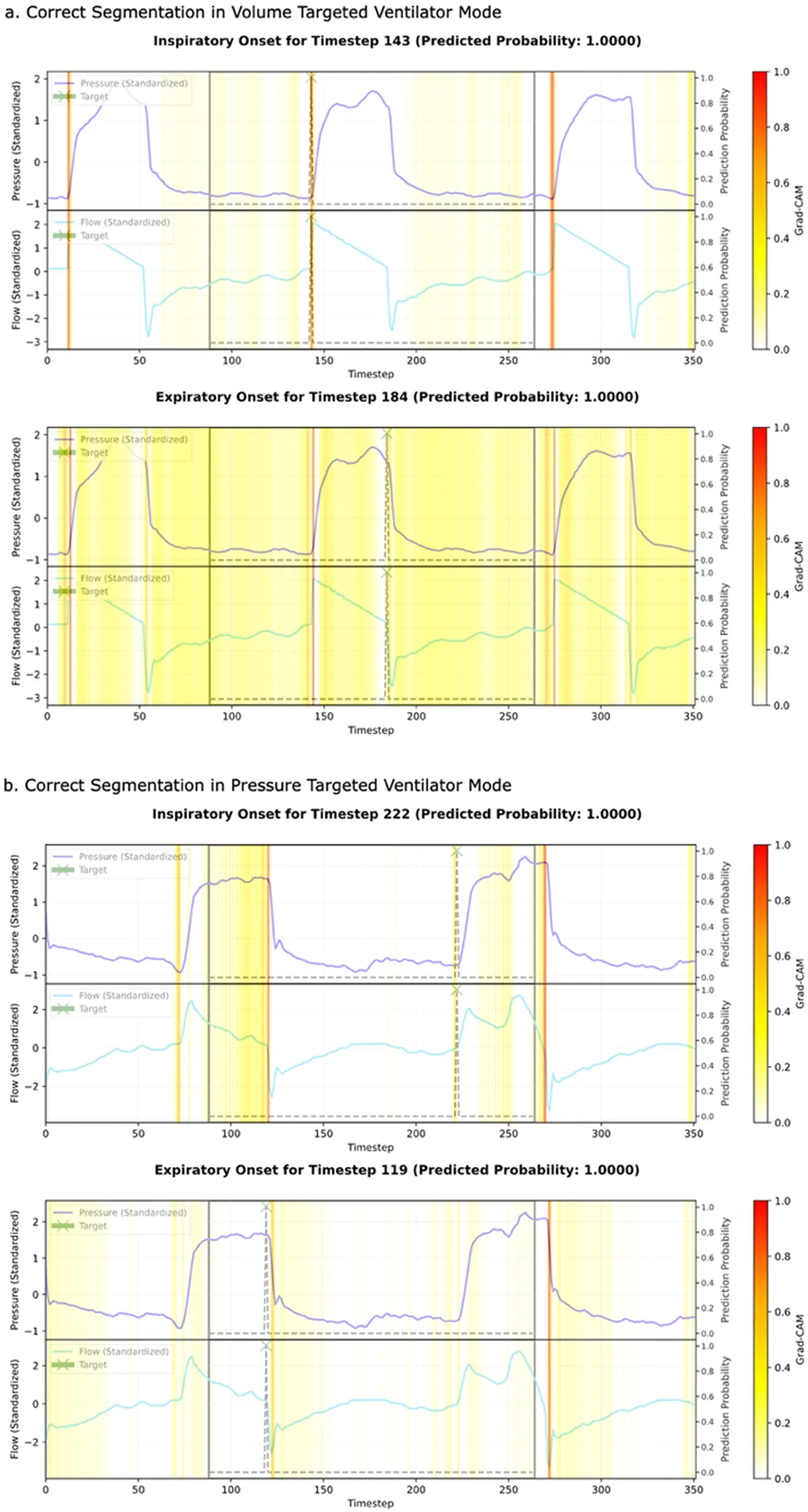

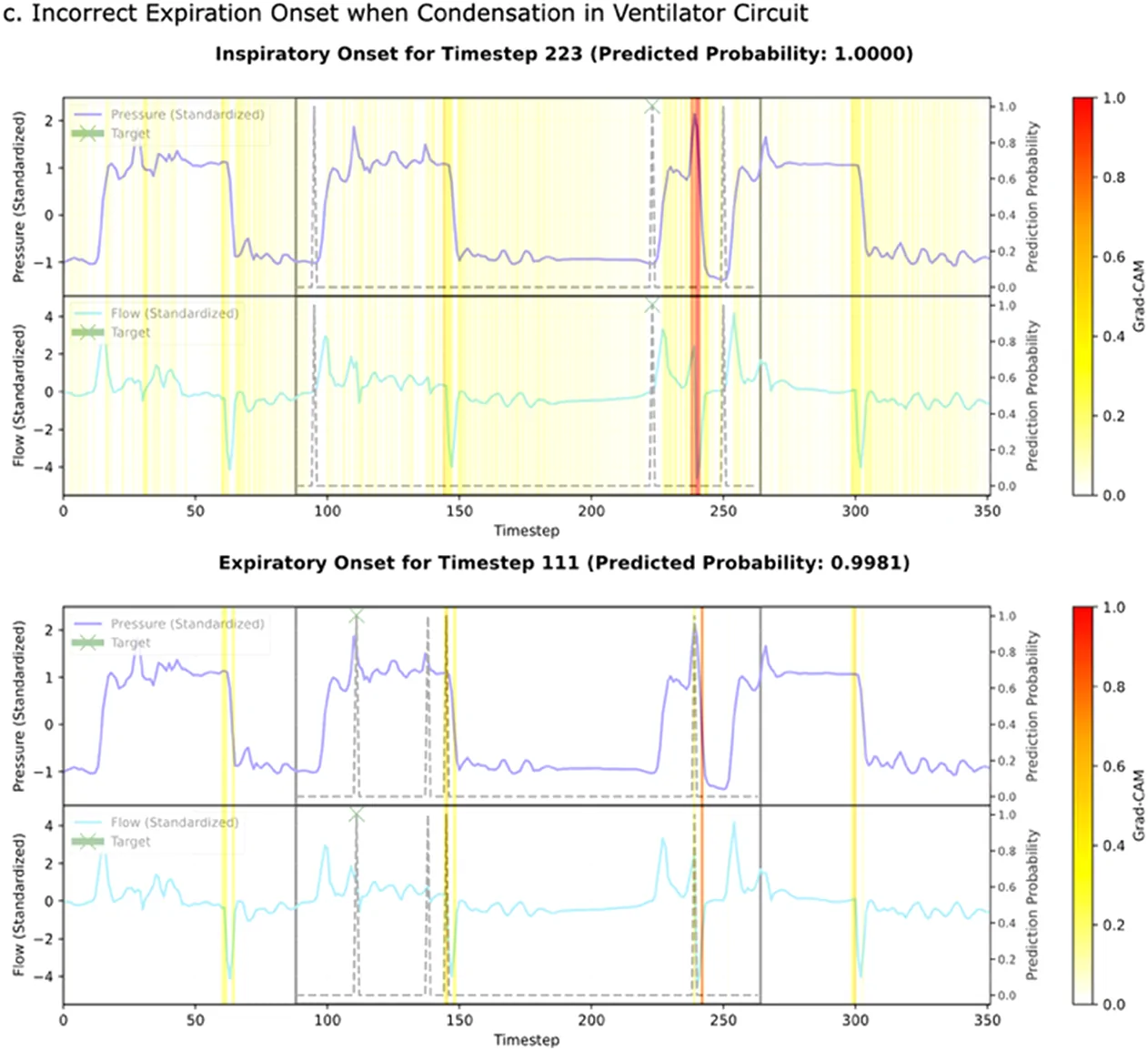
Gradient Weighted Class Activated Map. Red represents the highest activation values in the layer, which indicate regions of the waveform that had the greatest influence on the model’s prediction. The heat map reflects the model’s attention across both pressure and flow waveforms together. Each panel displays inspiratory onset detection (top) and expiratory onset detection (bottom). Dashed lines show the model’s predicted probability for each phase onset, and the ‘X’ marks the specific prediction referenced by the heat map. The area enclosed by solid black lines represents the output window, which is the time segment for which the model generated predictions, while the entire plot corresponds to the input window, which includes all the waveform data the model used to make those predictions. The final panel illustrates a correct inspiratory onset prediction (top) and an incorrect expiratory onset prediction (bottom), which is distinguished by minimal activation in the adjacent waveform regions.

The gradient-weighted class activation maps (Grad-CAM), applied to the last convolutional layer of the shared U-Net trunk, are illustrated in three manually selected input windows (Fig. 2). The central region of each plot represents the output window, and each plot depicts activation heatmaps for an onset prediction at a single timestep in the 1-D time series. Across the breaths, the inspiratory and expiratory onsets of neighboring breaths appear to influence segmentation, suggesting that the model may be learning from the periodicity of the breaths. The plot demonstrates that several regions of each window contribute to the overall prediction. The final panel shows an incorrect expiratory onset prediction, notable for its lack of activation in the surrounding time segments.

### Clinical application of the model

**Fig. 3:**
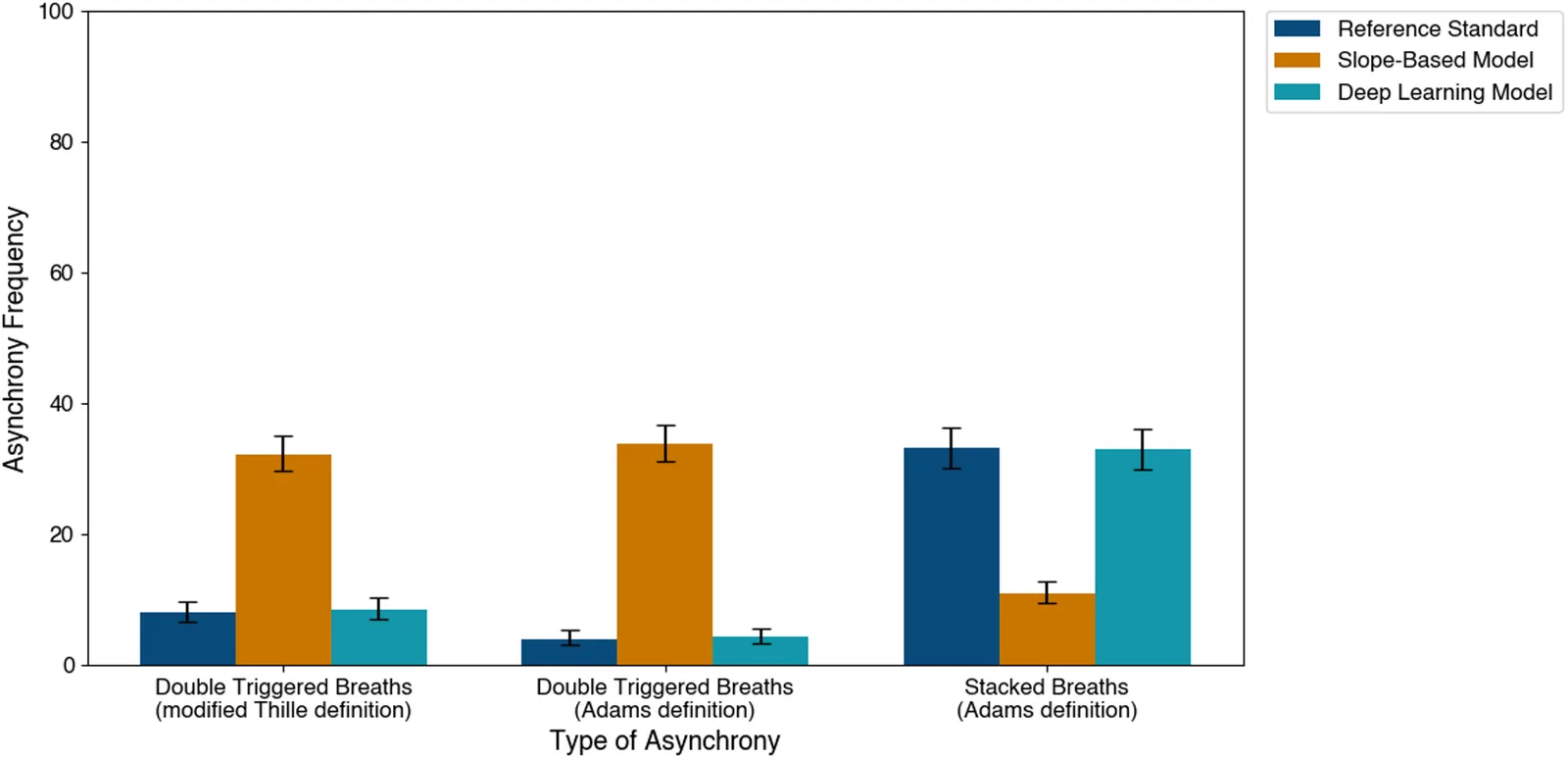
Comparison of asynchrony frequency identified by different segmentation models. Error bars represent 95% confidence intervals.

Finally, we applied the different segmentation approaches to quantify the frequency of asynchronous breathing patterns. Using the reference standard, the prevalence of double-triggered breaths was 8% under the Thille definition and 4% under the Adams definition, while stacked breaths under the Adams definition occurred in 33% of cases^12,22^. Use of the deep learning model’s inspiratory and expiratory onsets reproduced these frequencies with no statistically significant differences (double triggering: 9% by the Thille definition, *p* = 0.80; 4% by the Adams definition, *p* = 0.74; stacked: 33%, *p* = 0.91). In contrast, the derivative backtracking method yielded substantially different estimates (double triggering: 32% by the Thille definition, *p* < 0.01; 34% by the Adams definition, *p* < 0.01; stacked: 11%, *p* < 0.01) (Fig. 3).

## Discussion

In this study, we aimed to develop a deep learning model to accurately classify the onset of both inspiration and expiration in a real-world dataset of ventilator waveforms enriched for PVA. We found that our model outperformed traditional heuristic approaches for ventilator waveform segmentation in this dataset, with high precision and recall, and very few errors, maintaining performance even in asynchronous breaths. Notably, the model’s segmentations reproduced asynchrony frequencies that statistically matched those derived from the reference standard, which combined ventilator-identified inspiratory onsets and expert-annotated expiratory onsets. In contrast, rule-based approaches produced markedly different estimates, underscoring how methodological variability can drive heterogeneity in reported asynchrony rates across studies. Because PVAs are associated with adverse outcomes such as lung injury, diaphragm dysfunction, prolonged ventilation, and mortality, consistent definitions and reliable segmentation methods are essential to advance research and clinical monitoring^32^.

There is growing interest in using artificial intelligence to detect PVAs in near real time, but the success of such models depends heavily on the quality of training data and the reference standard labels used^32,33^. Our results demonstrate that in addition to PVA annotations, the segmentation of breaths themselves is a critical factor shaping PVA quantification. Prior reviews and a recent international expert consensus emphasize the lack of agreement in PVA definitions as a major barrier to reliable clinical tools^32–34^. Our findings show that consensus methods for breath segmentation should be established alongside consensus definitions of PVAs to support reproducible model development across institutions.

Although few deep learning methods have been published specifically for invasive mechanical ventilator breath segmentation, comparison of our results with related deep learning–based ventilator waveform and physiologic time-series segmentation studies provides context for expected model performance. In this regard, our high-performance results are consistent with those of Bakkes et al., whose conference paper also employed a U-Net architecture to identify physiologic features in ventilator waveforms^20^. Whereas their model was designed to detect the patient’s inspiratory and expiratory efforts, our approach focused on identifying phase transitions based on when the ventilator initiates and terminates breath delivery. These complementary perspectives are both necessary for detecting patient–ventilator asynchronies, which arise from mismatches between the patient’s efforts and the ventilator’s timing. We extend their work by demonstrating the versatility of the U-Net architecture for physiologic time-series analysis, using a dataset that included more than twice as many patients and breaths, as well as a substantial number of double-triggered breaths that were absent in their cohort.

While Bakkes et al.’s model showed discrepancies between the true incidence of PVA types (delayed inspiration, early cycling, late cycling, and ineffective efforts) and the incidence detected by their model, our model reproduced the PVA frequencies observed in the reference standard. This difference may reflect both the type of asynchronies evaluated—our model focused on double triggering and breath stacking, which display more pronounced waveform deflections—and methodological differences in how the reference standard was incorporated. Specifically, our model trained on exact onset time points, whereas their approach used a 210-millisecond onset window. That interval may be too broad, given that some of their PVA definitions involved timing differences of only 100–300 milliseconds between the patient’s respiratory effort and the ventilator’s corresponding termination of breath delivery. Additionally, in our data, when the inspiratory onset was detected more than 100 milliseconds after the true onset, the estimated tidal volume decreased by over 10%. Such timing errors could affect the detection of asynchronies that depend on tidal volume measurements, including some of those analyzed in our study. These findings underscores that precise onset detection is critical for accurately characterizing these physiologic events.

Our results are further supported by another study that applied a U-Net architecture to segment invasive physiologic signals, specifically the onset and offset of atrial activity in electrophysiologic recordings^35^. Although that model achieved slightly lower performance than ours, their model showed resilience even when artificial noise was introduced. This finding highlights the suitability and resilience of U-Net architectures for segmenting complex time-series physiologic data, even under noisy conditions, such as PVAs. More recently, Wang et al. proposed an unsupervised learning approach for breathing cycle–aware segmentation to support patient–ventilator asynchrony detection in non-invasive ventilation, demonstrating superior performance compared with multiple traditional heuristic and signal-processing-based segmentation techniques^36^. Despite differences in ventilation modality and segmentation granularity, these findings provide additional context suggesting that learning-based approaches offer advantages over purely heuristic or signal-processing methods for robust segmentation of ventilatory waveforms.

Automated segmentation addresses practical challenges in the field. Manual segmentation is time consuming and limits sample sizes, while ventilator-generated segmentation is inconsistently available across ventilator platforms and middleware solutions that collect ventilator waveform data. Heuristic-based approaches may fail because they typically use one waveform input (usually flow), which may differ across ventilator modes and PVA types. In contrast, Grad-CAM visualizations revealed that our model forms complex, temporally distributed feature maps across the breath and neighboring cycles, integrating information from both flow and pressure inputs to guide segmentation, which may explain our model’s consistent performance in both synchronous and asynchronous breaths. By providing a robust, automated solution, our model represents an important step down the path toward standardization of ventilator waveform data segmentation and subsequent research with this rich data type. The model is computationally efficient and enables clinically timely inference. These characteristics make it practical for bedside use, where continuous breath-by-breath segmentation could form the foundation for a unified model for the automated detection of PVAs.

This study has several limitations. External validation on additional ventilator types and at multiple centers is needed to assess broader generalizability. While our limited access to MV waveform data from a single health system and ventilator type prohibited external validation testing, we incorporated several regularization strategies, which may improve generalization. Nonetheless, future studies will need to confirm our results on independent datasets and diverse ventilator platforms. Additionally, our baseline heuristics were implemented according to published descriptions, but source code was not available, which may have limited the fidelity of reproduction. Comparisons with alternative segmentation approaches may further contextualize the model’s performance. The inspiratory onset reference standard was also proprietary, limiting transparency into how those labels were generated. Furthermore, the model’s segmentation performance should be systematically validated on additional types of PVAs, and commonly encountered artifacts such as cough or condensation in the circuit to ensure broad applicability, as manual review of mis-segmentations revealed challenges in detecting some cases. Finally, future work should evaluate the model’s actionability and its clinical impact when used to guide interventions aimed at reducing PVAs.

In conclusion, our attention-gated U-Net achieves high segmentation accuracy and sufficient computational efficiency for clinically timely use. By enabling data harmonization and scalable analysis, automated segmentation provides a foundation for more consistent study of ventilator–patient interactions. Because segmentation directly impacts PVA identification, future work should focus on standardizing not only PVA definitions but also breath segmentation methods, paving the way for precise, reproducible, and clinically deployable tools for automated PVA monitoring.

## Supplementary Information

Below is the link to the electronic supplementary material.

Supplementary Material 1.

## Data Availability

Data are available on reasonable request.

## Code availability

Code is available on reasonable request.
