# Supplementary material for: Deep learning for time-series segmentation of mechanical ventilator waveforms
Source: Sci Rep. 2026 Jun 28;16:29682. doi: 10.1038/s41598-026-58565-0 (PMC13600923; doi:10.1038/s41598-026-58565-0)
Supplement: Supplementary file 1 [file 41598_2026_58565_MOESM1_ESM.docx]

Supplementary Table 1. Checklist for Artificial Intelligence in Medical Imaging (CLAIM): 2024 Update

| **Section / Topic** | **No.** | **Item** | **Page / Line** | **No** | **NA** |
| --- | --- | --- | --- | --- | --- |
| **TITLE** / **ABSTRACT** |  |  |  |  |  |
|  | **1** | Identification as a study of AI methodology, specifying the category of technology used (e.g., deep learning) | **1** |  |  |
| **ABSTRACT** |  |  |  |  |  |
|  | **2** | Summary of study design, methods, results, and conclusions | **2** |  |  |
| **INTRODUCTION** |  |  |  |  |  |
|  | **3** | Scientific and/or clinical background, including the intended use and role of the AI approach | **3-4** |  |  |
|  | **4** | Study aims, objectives, and hypotheses | **4** |  |  |
| **METHODS** |  |  |  |  |  |
| ***Study Design*** | **5** | Prospective or retrospective study | **5** |  |  |
|  | **6** | Study goal | **10-11** |  |  |
| ***Data*** | **7** | Data sources | **5-6** |  |  |
|  | **8** | Inclusion and exclusion criteria | **5** |  |  |
|  | **9** | Data pre-processing | **5-6** |  |  |
|  | **10** | Selection of data subsets | **5** |  |  |
|  | **11** | De-identification methods | **5** |  |  |
|  | **12** | How missing data were handled | **5** |  |  |
|  | **13** | Image acquisition protocol | **5** |  |  |
| ***Reference Standard*** | **14** | Definition of method(s) used to obtain reference standard | **15** |  |  |
|  | **15** | Rationale for choosing the reference standard | **5-6** |  |  |
|  | **16** | Source of reference standard annotations | **5-6** |  |  |
|  | **17** | Annotation of test set | **5-6** |  |  |
|  | **18** | Measures of inter- and intra-rater variability of features described by the annotators |  |  | **x** |
| ***Data Partitions*** | **19** | How data were assigned to partitions | **6** |  |  |
|  | **20** | Level at which partitions are disjoint | **6** |  |  |
| ***Testing Data*** | **21** | Intended sample size | **6** |  |  |
| ***Model*** | **22** | Detailed description of model | **7-10** |  |  |
|  | **23** | Software libraries, frameworks, and packages | **6, 10** |  |  |
|  | **24** | Initialization of model parameters | **9** |  |  |
| ***Training*** | **25** | Details of training approach | **8-10** |  |  |
|  | **26** | Method of selecting the final model | **9-10** |  |  |
|  | **27** | Ensembling techniques |  |  | **x** |
| ***Evaluation*** | **28** | Metrics of model performance | **10** |  |  |
|  | **29** | Statistical measures of significance and uncertainty | **11** |  |  |
|  | **30** | Robustness or sensitivity analysis | **11** |  |  |
|  | **31** | Methods for explainability or interpretability | **11** |  |  |
|  | **32** | Evaluation on internal data | **10** |  |  |
|  | **33** | Testing on external data |  |  | **x** |
|  | **34** | Clinical trial registration |  |  | **x** |
| ***RESULTS*** |  |  |  |  |  |
| ***Data*** | **35** | Numbers of patients or examinations included and excluded | **12** |  |  |
|  | **36** | Demographic and clinical characteristics of cases in each partition |  |  | **x** |
| ***Model performance*** | **37** | Performance metrics and measures of statistical uncertainty | **12-15** |  |  |
|  | **38** | Estimates of diagnostic performance and their precision | **17-18** |  |  |
|  | **39** | Failure analysis of incorrect results | **15-17** |  |  |
| ***DISCUSSION*** |  |  |  |  |  |
|  | **40** | Study limitations | **21-22** |  |  |
|  | **41** | Implications for practice, including intended use and/or clinical role | **21** |  |  |
| ***OTHER INFORMATION*** |  |  |  |  |  |
|  | **42** | Provide a reference to the full study protocol or to additional technical details |  | **x** |  |
|  | **43** | Statement about the availability of software, trained model, and/or data | **22** |  |  |
|  | **44** | Sources of funding and other support; role of funders | **22** |  |  |

Supplementary Table 2. Ventilator Modes in the Dataset Identified by a Machine Learning Classifier

| Mode* | Prevalence |
| --- | --- |
| Pressure Control | 4,680 breaths (52.6%) |
| Volume Control | 1,789 breaths (20.1%) |
| Pressure Support | 1,749 breaths (19.7%) |
| Proportional Assist Ventilation | 679 breaths (7.6%) |
| Continuous Positive Airway Pressure | 0 breaths (0%) |

* This classifier requires a rolling window of 10 breaths to generate classifications, so the first 10 breaths of each file did not generate a prediction (9.4% of breaths in the dataset).

Supplementary Table 3. Results of Per Patient Model Performance in the Test Set

|  | Inspiratory Onset F1 Score (within 0.1s) | Expiratory Onset F1 Score (within 0.1s) |
| --- | --- | --- |
| Patient 1  (n = 299 breaths) | 0.998 | 0.983 |
| Patient 2  (n = 300 breaths) | 0.995 | 0.988 |
| Patient 3  (n = 300 breaths) | 0.995 | 0.997 |
| Patient 4  (n = 300 breaths) | 0.997 | 0.987 |
| Mean ± Standard Deviation | 0.996 ± 0.001 | 0.989 ± 0.005 |

Supplementary Table 4. Validation-set performance of selected CNN architecture variants evaluated during model development

|  | Validation Set | | | |
| --- | --- | --- | --- | --- |
|  | Inspiratory Onset | | Expiratory Onset | |
| Model | F1 | F1 (within 0.1s) | F1 | F1 (within 0.1s) |
| Attention-gated U-Net (hybrid loss, 352-step input) | 0.811 | 0.969 | 0.944 | 0.970 |
| Attention-gated U-Net (hybrid loss, 176-step input) | 0.792 | 0.961 | 0.922 | 0.975 |
| Standard U-Net (hybrid loss, 176-step input) | 0.768 | 0.943 | 0.912 | 0.964 |
| Attention-gated U-Net (Dice loss, 176-step input) | 0.790 | 0.937 | 0.882 | 0.965 |
| Attention-gated U-Net (Focal loss, 176-step input) | 0.334 | 0.808 | 0.628 | 0.801 |

a. Normal Volume Control Breath

b. Breath with Condensation in the Circuit

c. Delayed Cycling Asynchrony

d. Double Triggered Breath

e. Cough

Supplementary Figure 1. Examples of Waveforms in the Dataset.

Supplementary Figure 2. Change in median inspiratory tidal volume with increasing offset in inspiratory onset timing. The blue line represents the median calculated tidal volume when inspiratory onset is adjusted by the number of timesteps (each 0.02 seconds) on the x-axis. The dashed lines represent 1%, 2%, 5% and 10% decrease in median calculated tidal volume.
